# Generation of double knockout cattle via CRISPR-Cas9 ribonucleoprotein (RNP) electroporation

**DOI:** 10.1186/s40104-023-00902-8

**Published:** 2023-08-06

**Authors:** Gyeong-Min Gim, Kyeong-Hyeon Eom, Dong-Hyeok Kwon, Dae-Jin Jung, Dae-Hyun Kim, Jun-Koo Yi, Jae-Jung Ha, Ji-Hyun Lee, Seong-Beom Lee, Woo-Jae Son, Soo-Young Yum, Won-Wu Lee, Goo Jang

**Affiliations:** 1 Laboratory of Theriogenology and Biotechnology, Department of Veterinary Clinical Science, College of Veterinary Medicine and the Research Institute of Veterinary Science, Seoul National University, Seoul, Republic of Korea; 2 LARTBio Co., Ltd., Seoul, Republic of Korea; 3 Gyeongsangbukdo Livestock Research Institute, Yeongju, Republic of Korea; 4 Comparative Medicine Disease Research Center, Seoul National University, Seoul, Republic of Korea

**Keywords:** Beta-lactoglobulin, Cattle, CRISPR-Cas9, Electroporation, Knockout, MSTN, PRNP

## Abstract

**Background:**

Genome editing has been considered as powerful tool in agricultural fields. However, genome editing progress in cattle has not been fast as in other mammal species, for some disadvantages including long gestational periods, single pregnancy, and high raising cost. Furthermore, technically demanding methods such as microinjection and somatic cell nuclear transfer (SCNT) are needed for gene editing in cattle. In this point of view, electroporation in embryos has been risen as an alternative.

**Results:**

First, editing efficiency of our electroporation methods were tested for embryos. Presence of mutation on embryo was confirmed by T7E1 assay. With first combination, mutation rates for *MSTN* and *PRNP* were 57.6% ± 13.7% and 54.6% ± 13.5%, respectively. In case of *MSTN*/*BLG*, mutation rates were 83.9% ± 23.6% for *MSTN*, 84.5% ± 18.0% for *BLG*. Afterwards, the double-KO embryos were transferred to surrogates and mutation rate was identified in resultant calves by targeted deep sequencing. Thirteen recipients were transferred for *MSTN*/*PRNP*, 4 calves were delivered, and one calf underwent an induction for double KO. Ten surrogates were given double-KO embryos for *MSTN*/*BLG*, and four of the six calves that were born had mutations in both genes.

**Conclusions:**

These data demonstrated that production of genome edited cattle via electroporation of RNP could be effectively applied. Finally, *MSTN* and *PRNP* from beef cattle and *MSTN* and *BLG* from dairy cattle have been born and they will be valuable resources for future precision breeding.

**Supplementary Information:**

The online version contains supplementary material available at 10.1186/s40104-023-00902-8.

## Introduction

Genome editing has played a powerful role in various fields. In the same line with other fields, in livestock, genome editing tools such as ZFN, TALENs, and CRISPR/Cas9, has been applied to disease resistance, climate response, allergy free and increasing productivity [1–8]. Recently, several countries including Japan, Brazil and Australia announced that genome edited livestock with simple indel mutation was not categorized into genetically modified organisms (GMOs) because of no integration of the exogenous DNA. In USA, FDA announce that genome edited cattle will be approved to be low level risk [9]. It is believed that in response to these changes, the development of gene-editing cattle is predicted to be accelerated for improving the traits.

However, genome editing progress in cattle hasn’t been fast compared to other mammal species in that there are some issues including long gestational periods, single pregnancy, and high cost. And high skilled person with microinjection and somatic cell nuclear transfer (SCNT) technologies are needed to produce the genetically engineered cattle to date. Particularly, while SCNT with high frequency mutated somatic cells have contributed to similar embryonic developmental competence with in vitro fertilized embryos, abnormal reprogramming issues such as embryonic absorption and sudden death enable us to be hard for progress [10, 11]. Thus, to produce a live cloned offspring, many surrogate mothers are needed [3] compared to using in vitro fertilization combined with microinjection, which are reported in our paper [1, 12].

And some studies, as an alternative for microinjection, electroporation showed that it can be used for knockout animal with efficient and simple way [13–15]. However, although there have been in vitro studies on electroporation in cattle [13, 14], in vivo results have not yet been demonstrated. Here, we proved that double gene edited cattle were efficiently born by electroporation via ribonucleoprotein (RNP).

## Methods

### In vitro embryo production

In vitro oocytes were collected in the ovaries from beef or dairy slaughterhouses. Immature oocytes were matured in TCM based medium as previously reported [1]. Motile spermatozoa were selected using the Percoll gradient method as previous described. Briefly, frozen-thawed semen from F0 bull at 35 ºC was filtered by centrifugation on a Percoll discontinuous gradient (45%–90%) at 366 × *g* for 15 min. To produce the 45% Percoll solution, 1 mL of capacitation-Tyrode's albumin lactate pyruvate (TALP) medium was added to 1 mL of 90% Percoll. The sperm pellet was washed two times by the addition of 3 mL of the capacitation-TALP medium and was subsequently centrifuged at 366 × *g* for 5 min. Washed motile spermatozoa were used for IVF. Spermatozoa (1–2 × 10^6^ sperm/mL) were incubated with mature oocytes for 18 h in 50 μL microdrops of IVF-TALP medium covered with mineral oil (Nidacon, Cat. no. NO-100) in a humidified atmosphere of 5% CO_2_ at 38.5 ºC. After 18 h of co-incubation, cumulus cells were removed from presumptive zygotes. The zygotes were cultured in a two-step chemically defined culture media [1, 12] that was covered in mineral oil in an atmosphere of 5% O_2_, 5% CO_2_, and 90% N_2_ at 38.5 °C.

### Designing sgRNA and gene mutation assay

Single guide RNA (sgRNA) targeting bovine *PRNP* (exon3), *MSTN* (exon2) and *BLG* (exon3) was designed by Cas-Designer software (http://www.rgenome.net/cas-designer/) that showed sgRNA candidates for the target genome (Additional file 1) [1, 13]. Following the details of kit manual, the sgRNA was synthesized using Precision gRNA synthesis kit (ThermoFisher, A29377).

Gene mutation was confirmed through the T7 endonuclease 1 (T7E1) assay. For this, genome DNA was extracted by kit (Qiagen, 69504). The PCR primers (Additional file 1) for target loci (*PRNP*, *MSTN*, and *BLG*) was designed using PRIMER3 software (http://bioinfo.ut.ee/primer3-0.4.0), and the target sequence was amplified by polymerase chain reaction (PCR) at 94 ºC for 5 min, 35–40 cycles at 94 ºC for 20 s, at 57 ºC for 30 s, at 72 ºC for 35 s, and 72 ºC for 5 min. The PCR product from each sample was treated with T7E1 enzyme (NEB, Cat. No. M0302L) to detect gene mutations. In case of single-cell analysis, wildtype PCR product was added into all samples to detect homozygous mutation. Digested and undigested mixes were observed on a 1% agarose gel. The estimated gene modification was calculated as described previously [16].

### Electroporation of RNP

Genome Editor electroporator (BEX, GEB 15) and electrode (gap: 1.0 mm, volume: 40 µL) (BTX, 45–0104) were used for electroporation. The electrode was connected to the electroporator and was set under a stereoscopic microscope. Before electroporation, bovine zygotes were washed with Opti-MeM l (ThermoFisher, 31985062). At one time, 30–40 bovine zygotes were electroporated. Zygotes 18 h after IVF were washed with Opti-MEM I three times to remove the serum in the medium, placed in a line in the electrode gap filled with 10 µL of Opti-MEM I which is containing 200 ng/ µL of Cas9 protein (ThermoFisher, A36499) and 100 ng/µL each sgRNA, and subjected to electroporation. The electroporation condition was 15 V (3 ms ON + 97 ms OFF) × 7 times. After electroporation, the zygotes were immediately collected from the electrode chamber and subjected to four washes with TCM-199 based medium followed. The embryos were then cultured in chemically defined medium at 38.5 °C, 5% CO_2_, and 5% O_2_ in an incubator.

### Primary cell culture and genomic DNA extraction

Primary cells were obtained by biopsy of the ear skin of calves. The ear skin was chopped into small pieces with a sterile scalpel and then washed several times and incubated at 38.5 °C for 4–18 h in Hank's Balanced Salt Solution (Gibco, 14175095) supplemented with collagenase (Collagenase type I, Gibco, Cat. no. 17–100–017). The following day the dispersed cells were washed several times in DMEM (Gibco, 21068028) and cultured in DMEM supplemented with 10% fetal calf serum (Gibco, GIB-11150–059), 1% penicillin/streptomycin (Gibco, Cat. no. 15140148), 1% non-essential amino acids (Gibco, 11140050), and 100 mmol/L β-mercaptoethanol (Sigma–Aldrich, M3418). Genomic DNA from primary cells was extracted using a DNA extraction kit (Qiagen, 69504). Extracted DNA was used for PCR-T7E1 assay, Cloning-Sanger sequencing and deep sequencing.

### Deep sequencing

Target sites were first amplified to a size of ~1000 bp from extracted genomic DNA using Maxime™ PCR PreMix i-StarTaq (Intron biotechnology, Cat. 25167). The 1^st^ PCR amplicons were amplified again to a size of ~220 bp through 2^nd^ primers having custom index sequence. And 2^nd^ PCR products also were amplified for adding adaptor sequence to NGS (Mini-seq, Illumina). Primers used in this study are listed in Additional file 2.

Then, 3^rd^ PCR amplicons were pooling and purified using a PCR purification kit (Macherey-Nagel™ NucleoSpin®, Gel and PCR clean up, Cat. 740609). This purified library was used for NGS according to the illumina manual. The sequencing results of Mini-seq was saved as fastaq files and it could be analyzed through Cas-analyzer (www.rgenome.net).

## Results

### In vitro production of double gene edited embryos via RNP electroporation

**Fig. 1:**
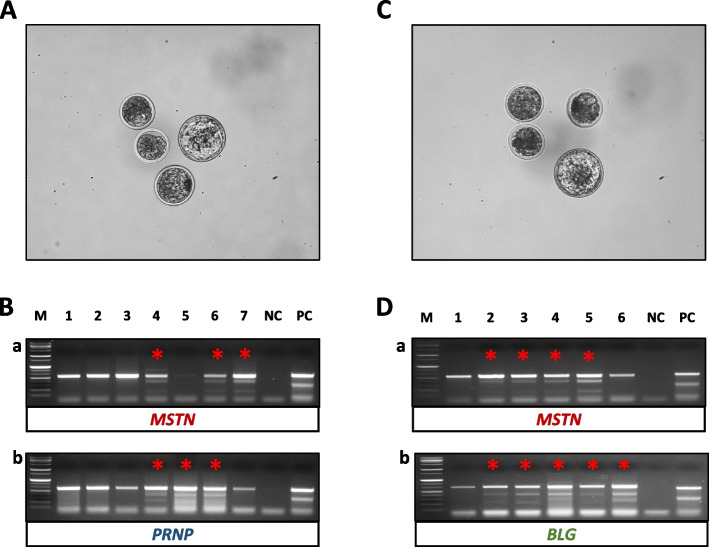
In vitro production of double gene edited embryo via RNP electroporation. **A** *MSTN*/*PRNP* double gene edited embryos. **B** T7E1 assay results for mutation of *MSTN* (a) and *PRNP* (b) in *MSTN*/*PRNP* double gene edited embryos (lane 2–7). **C** *MSTN*/*BLG* double gene edited embryos. **D** T7E1 assay results for mutation of *MSTN* (a) and *BLG* (b) in *MSTN/BLG* double gene edited embryos (lane 2–6). M = 1 kb ladder, lane 1 = Wild type, *N* = negative control, *P* = positive control, red asterisk = lane with T7E1 positive results

*MSTN/PRNP* double knockout was performed three times using 320 embryos, and the development competence of blastocysts was 16.2% ± 2.8%. To analyze the mutation of the target genes, individual blastocysts were analyzed by T7E1 assay. *MSTN* mutation was observed in 13 blastocysts (57.6% ± 13.7%) and *PRNP* mutation was observed in 12 blastocysts (54.6% ± 13.5%) (Fig. 1A and B). Among them, 45.1% ± 19.5% of the embryos showed positive results for both targets. In the case of *MSTN*/*BLG*, which was tested four times, and 232 in vitro fertilized embryos were used, and the formation rate of blastocysts was 14.0% ± 4.2%. Among them, 16 blastocysts showed *MSTN* mutation (83.9% ± 23.6%), and 17 blastocysts showed *BLG* mutation (84.5% ± 18.0%) (Fig. 1C and D). In *MSTN*/*BLG*, 80.4% ± 24.3% were double positive.

### Production of double gene edited cattle

Based on mutation in vitro produced embryos, one blastocyst per a recipient was transferred, and presumptive *MSTN*/*PRNP* knockout embryos were transplanted into a total of 13 recipients, and presumptive *MSTN*/*BLG* knockout embryos were transplanted into 10 recipients. When the 60-day evaluation was carried out, pregnancy was confirmed in 14 out of 23. A total of 10 calves were born from 14 pregnant individuals. Finally, four calves from *MSTN*/*PRNP* group and six calves from *MSTN*/*BLG* group were born.

**Fig. 2:**
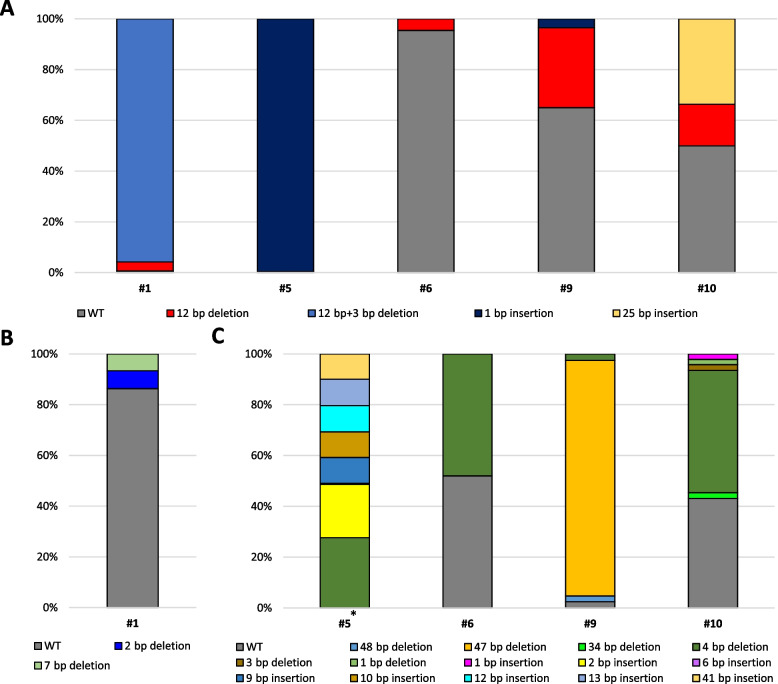
Ratio of mutation patterns in each target loci.** A** Mutation pattern of *MSTN* gene. **B** Mutation pattern in *PRNP* gene. **C** Mutation pattern in *BLG* gene. ^*^In calf #5, patterns were highly mixed each other for *BLG*. Only relative ratio of patterns is shown.

To evaluate the mutation of the target genes (*MSTN*, *PRNP* and *BLG*), genomic DNA derived from skin tissue were analyzed by targeted deep sequencing. *MSTN*/*PRNP* mutation was observed in one calf and *MSTN*/*BLG* mutation were observed in 4 calves (Table 1). Types of alleles edited for each target loci are summarized in Fig. 2. Additionally, to clarify the efficiency of double knock-out for target genes, we checked mutation rate of each target genes in single cell level by T7E1 assay, using primary cells from 5 calves in which we confirmed presence of mutation (Table 2). In *MSTN*/*PRNP* mutated cattle, 78.38% of cells showed *MSTN* mutation and 24.32% of cells showed *PRNP* mutation with only 2.7% of cells showed *MSTN*/*PRNP* double mutation. In case of *MSTN*/*BLG* mutated calves, all tested cells showed *BLG* mutation with variable rate of *MSTN* mutation (Table 2).

**Table 1:** Deep sequencing results for mutation rates of double target genes in individual calves

**ID**	**Beef**		**Dairy**		**Sex**	**Age**
	**MSTN**	**PRNP**	**MSTN**	**BLG**		
#1	99.3%	13.7%			Male	10 months
#2	-	-			Male	-
#3	-	-			Female	-
#4	-	-			Male	-
#5^*^			99.6%	100%	Female	Died due to infection
#6			4.6%	48.0%	Female	10 months
#7^**^			-	-	Female	Died after birth
#8			0.9%	0%	Male	-
#9			34.9%	97.6%	Female	8 months
#10			50.3%	55.3%	Male	8 months

^*^#5 was died and diagnosed as bacterial infection; ^**^After autopsy, there is no finding any pathological infection and congenital defects. #1–#4: Beef cattle (MSTN/PRNP), #5–#10: Dairy cattle (MSTN/BLG), values below 0.1% were not presented. If there were various pattern, ratio of mutation pattern was presented

**Table 2:** Double mutation rate in single cell level

ID	Beef		Dairy		Double mutation
	**MSTN**	**PRNP**	**MSTN**	**BLG**	
#1	78.38% (29/37)	24.32% (9/37)	-	-	2.7% (1/37)
#5	-	-	80% (12/15)	100% (15/15)	80% (12/15)
#6	-	-	0% (0/21)	100% (21/21)	0% (0/21)
#9	-	-	45% (9/20)	100% (20/20)	45% (9/20)
#10	-	-	100% (20/20)	100% (20/20)	100% (20/20)

To figure out off-target effect of RNP on genome, the candidate sites were analyzed for presence of mutation on the sites using targeted deep sequencing and the results showed in Additional file 3.

**Fig. 3:**
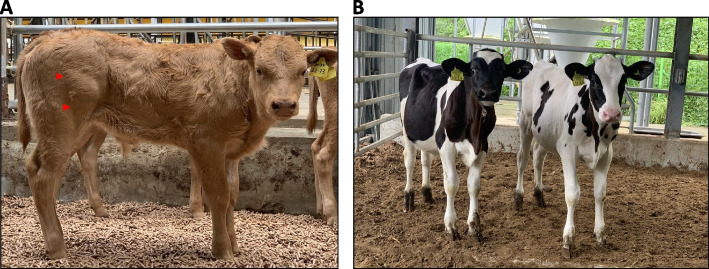
Representative pictures of double gene edited cattle. **A** *MSTN/PRNP* double gene edited beef cattle at current age (3-month-old). **B** *MSTN*/*BLG* double gene edited dairy cattle (4-month-old). Red arrow = enlarged biceps femoris

And blood analysis was conducted to monitor if any health problems were caused by environmental factors and/or genome editing. There were not any significant changes in blood analysis and no clinical symptoms were identified up to date (Additional file 4). Current pictures of calves are showed in Fig. 3.

## Discussion

Since genome editing have been applied to eukaryotic organisms, there were big changes in biological system [17]. In agriculture fields, genome editing rapidly has been applied to plants and livestock [18]. Particularly, cattle in livestock have been important species [19]. Genome edited cattle will be used for productivity, diseases resistance, and bioreactors [20]. In this study, to improve the genetic traits, genome editing technologies may be selected, over than one genetic trait. Among methods to produce genome-edited live animal, we selected electroporation method for its simplicity. Although there are always concerns about mosaicism when editing embryos, we tried to minimize mosaicism by using RNP complex, i.e., Cas9 protein and guide RNA complex according to previous report [7, 21].Three targeted loci were selected, that is, *MSTN* for productivity, *PRNP* for disease resistance, and BLG for allergy free milk. For beef cattle, *MSTN*/*PRNP* mutated calves were born and for dairy cattle, *MSTN*/*BLG* mutated calves were born via RNP electroporation. Currently, we analyzed genotyping from all the double knockout calves (Table 1) and in *MSTN*, where its phenotyping can be easily detected, it began to be observed (Fig. 3). Other phenotyping will be analyzed later because *BLG* should be analyzed after pregnancy and for *PRNP*, its test should be evaluated in the continuously follow up.

As summarized in Fig. 2, there was a unique fact in the genotyping analysis. In previous study, the *MSTN* mutated cattle showed only −12 bps [1]. However, in this study, although another gene delivery was applied into zygotes via RNP, −12 bps genotyping was dominantly identified as well, and new genotyping (1, 25 bps insertion and 3 bps deletion) was observed. In more details, no more than 2 genotyping was identified in calves with *MSTN* mutations (Fig. 2). However, three genotyping were observed in *PRNP* locus, and 3 or more mutations were observed in *BLG* except for one mutated calf. According to our results so far, −12 bp is dominantly observed in *MSTN* knockout cattle through microinjection or electroporation on IVF embryos. In the future, we will continue to monitor the results of knockout genotyping in vivo results through various gene loci. And we found very low percentage of off-target mutation in #5 (Additional file 3). After the offspring become at over than 12 months old, the genotyping and off-target analysis in their germ cells will be followed.

One interesting point is about double knockout event. In dairy breed calves, in which *MSTN* and *BLG* were targeted, there were high double knockout efficiency in randomly selected single cell colony analysis. However, in beef breed *MSTN/PRNP* KO calf, double knockout event in the offspring was very low while its event was comparable with that of *MSTN/BLG* in blastocyst stage. Because there is only one offspring with *MSTN/PRNP* KO, it is difficult to determine whether this occurrence was caused by specific target gene locus or breed/individual predisposition. We intend to conduct relevant research in the near future.

Recently, a genome edited organism without any recombinant DNA integration can be classified as non-GMO in many countries [9]. For instance, in Japan, *MSTN* edited fish is approved to be edible as a food chain [21] and in USA, low-risk determination was made for marketing genome-edited cattle [22]. In the case of animals, safety analysis is not necessary, and their health can be considered an indicator of animal product safety [23]. We were able to find no special complications or significant changes in blood analysis when we assessed the health of animals born as double knockouts, indicating that they are currently growing healthily. As a result, it is expected that the safety of meat and milk from these sources will be unaffected in the future.

## Conclusions

In conclusion, these data demonstrated that genome editing on bovine embryos via electroporation of RNP could be effectively applied and proved. Finally, beef cattle with *MSTN* and *PRNP* mutation and dairy cattle with *MSTN* and *BLG* mutation have been born and they will be valuable resources for future precise breeding.

## Supplementary Information

**Additional file 1. **List of guide RNA and detecting PCR primer sequences for each target genes.

**Additional file 2. **List of primer sequences for deep sequencing of each target genes and off-target sites.

**Additional file 3. **Off-target effect detection for gene edited calves.

**Additional file 4. **Blood analysis of calves with gene mutation.

## Data Availability

The datasets used and/or analyzed during the current study are available from the corresponding author on reasonable request.

## Abbreviations

BLG: Beta-lactoglobulin
Cas9: CRISPR associated protein 9
CRISPR: Clustered regularly interspaced short palindromic repeats
GMO: Genetically modified organism
IVF: In vitro fertilization
KO: Knock-out
MSTN: Myostatin gene
PRNP: Prion protein gene
RNP: Ribonucleoprotein
SCNT: Somatic cell nuclear transfer
sgRNA: Single guide RNA
T7E1: T7 endonuclease I
TALEN: Transcription activator-like effector nucleases
TALP: Tyrode’s albumin lactate pyruvate
ZFN: Zinc-finger nuclease
