# Supplementary material for: Generation of double knockout cattle via CRISPR-Cas9 ribonucleoprotein (RNP) electroporation
Source: J Anim Sci Biotechnol. 2023 Aug 6;14:103. doi: 10.1186/s40104-023-00902-8 (PMC10404370; doi:10.1186/s40104-023-00902-8)
Supplement: Supplementary file 1 — Additional file 1. List of guide RNA and detecting PCR primer sequences for each target genes. [file 40104_2023_902_MOESM1_ESM.pptx]

## Slide 1
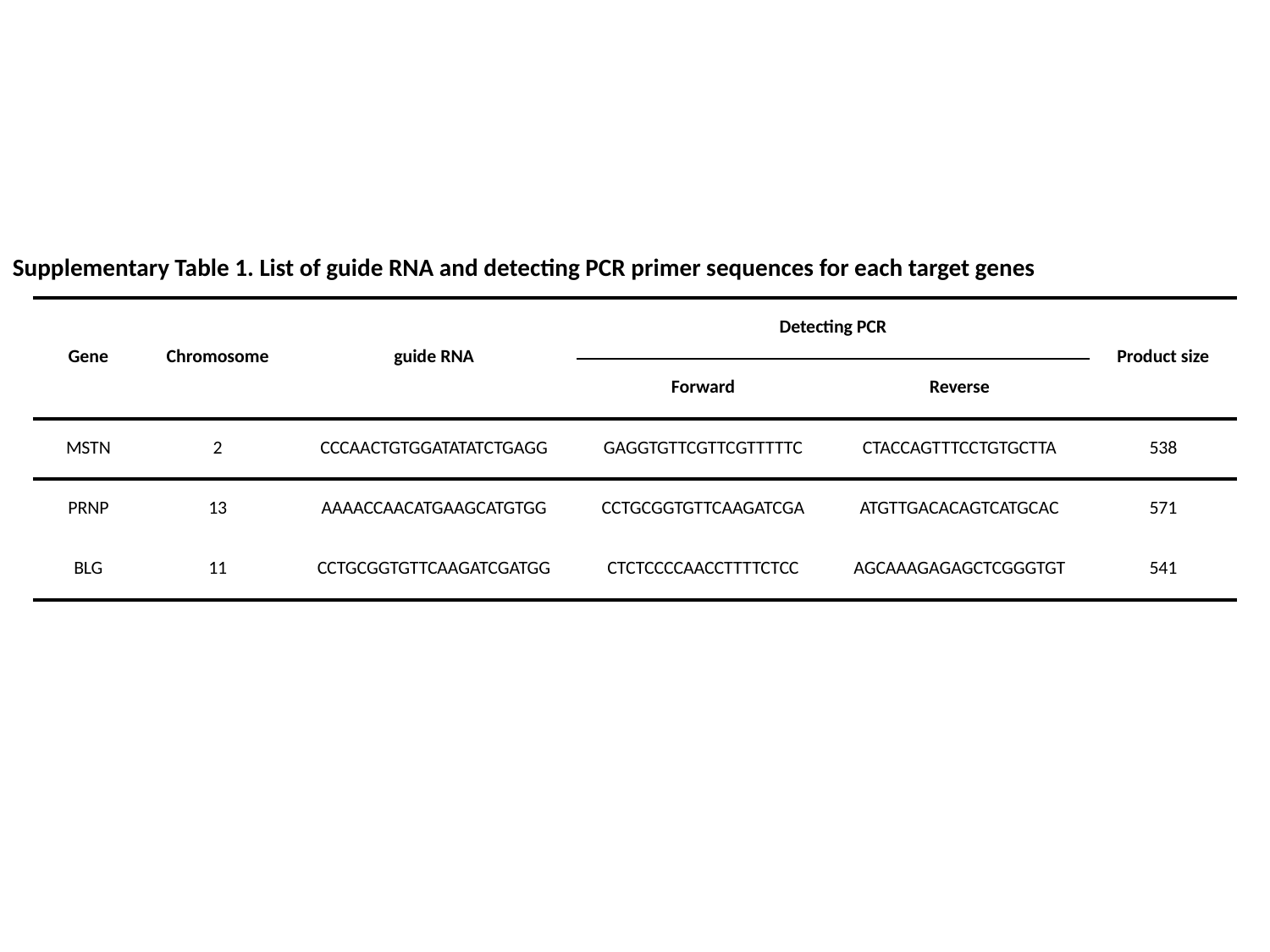

Supplementary Table 1. List of guide RNA and detecting PCR primer sequences for each target genes
| Gene | Chromosome | guide RNA | Detecting PCR | | Product size |
| --- | --- | --- | --- | --- | --- |
| | | | Forward | Reverse | |
| MSTN | 2 | CCCAACTGTGGATATATCTGAGG | GAGGTGTTCGTTCGTTTTTC | CTACCAGTTTCCTGTGCTTA | 538 |
| PRNP | 13 | AAAACCAACATGAAGCATGTGG | CCTGCGGTGTTCAAGATCGA | ATGTTGACACAGTCATGCAC | 571 |
| BLG | 11 | CCTGCGGTGTTCAAGATCGATGG | CTCTCCCCAACCTTTTCTCC | AGCAAAGAGAGCTCGGGTGT | 541 |
