# Supplementary material for: Generation of double knockout cattle via CRISPR-Cas9 ribonucleoprotein (RNP) electroporation
Source: J Anim Sci Biotechnol. 2023 Aug 6;14:103. doi: 10.1186/s40104-023-00902-8 (PMC10404370; doi:10.1186/s40104-023-00902-8)
Supplement: Supplementary file 2 — Additional file 2. List of primer sequences for deep sequencing of each target genes and off-target sites. [file 40104_2023_902_MOESM2_ESM.pptx]

## Slide 1
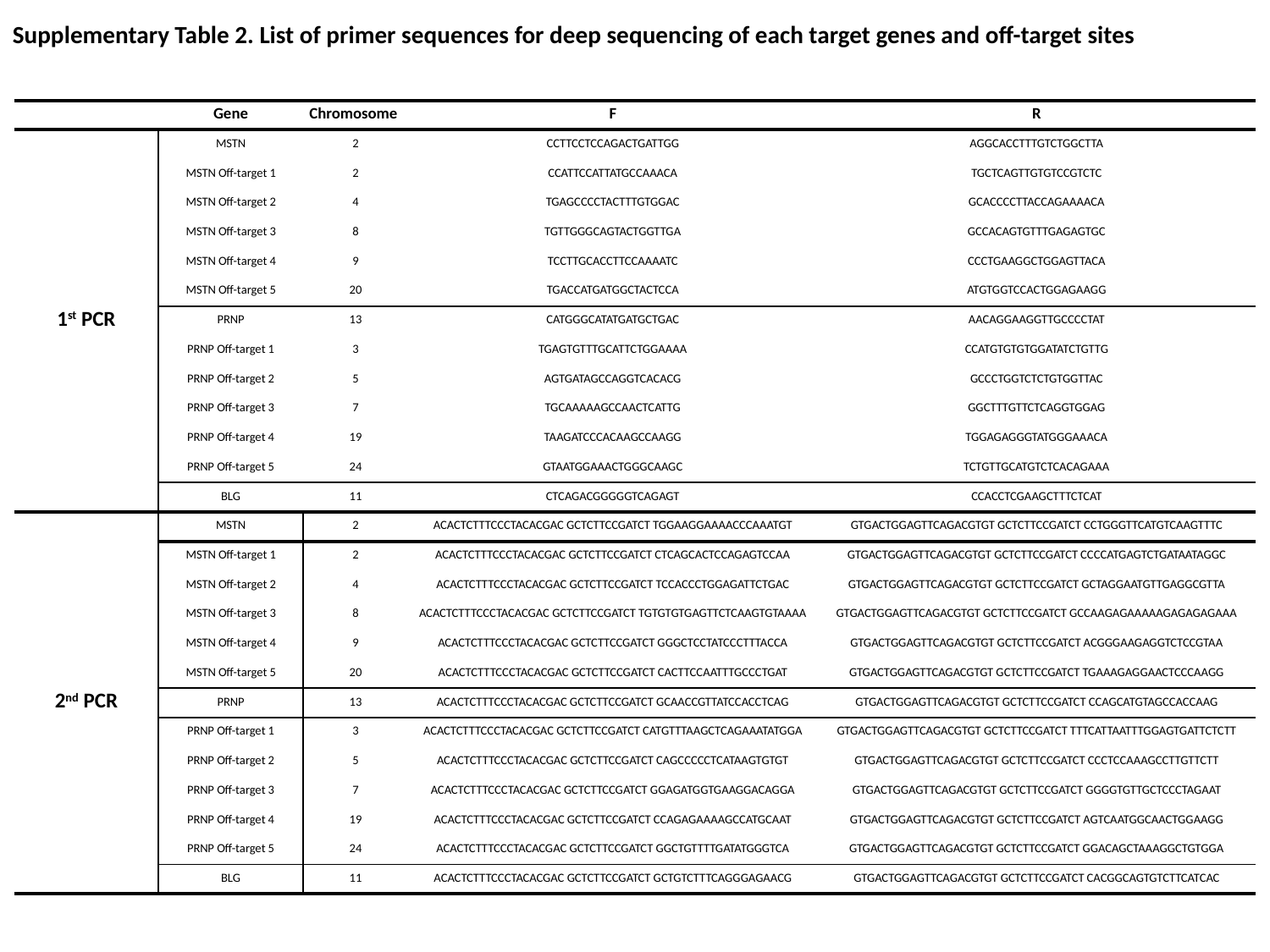

Supplementary Table 2. List of primer sequences for deep sequencing of each target genes and off-target sites
| | Gene | Chromosome | F | R |
| --- | --- | --- | --- | --- |
| 1st PCR | MSTN | 2 | CCTTCCTCCAGACTGATTGG | AGGCACCTTTGTCTGGCTTA |
| | MSTN Off-target 1 | 2 | CCATTCCATTATGCCAAACA | TGCTCAGTTGTGTCCGTCTC |
| | MSTN Off-target 2 | 4 | TGAGCCCCTACTTTGTGGAC | GCACCCCTTACCAGAAAACA |
| | MSTN Off-target 3 | 8 | TGTTGGGCAGTACTGGTTGA | GCCACAGTGTTTGAGAGTGC |
| | MSTN Off-target 4 | 9 | TCCTTGCACCTTCCAAAATC | CCCTGAAGGCTGGAGTTACA |
| | MSTN Off-target 5 | 20 | TGACCATGATGGCTACTCCA | ATGTGGTCCACTGGAGAAGG |
| | PRNP | 13 | CATGGGCATATGATGCTGAC | AACAGGAAGGTTGCCCCTAT |
| | PRNP Off-target 1 | 3 | TGAGTGTTTGCATTCTGGAAAA | CCATGTGTGTGGATATCTGTTG |
| | PRNP Off-target 2 | 5 | AGTGATAGCCAGGTCACACG | GCCCTGGTCTCTGTGGTTAC |
| | PRNP Off-target 3 | 7 | TGCAAAAAGCCAACTCATTG | GGCTTTGTTCTCAGGTGGAG |
| | PRNP Off-target 4 | 19 | TAAGATCCCACAAGCCAAGG | TGGAGAGGGTATGGGAAACA |
| | PRNP Off-target 5 | 24 | GTAATGGAAACTGGGCAAGC | TCTGTTGCATGTCTCACAGAAA |
| | BLG | 11 | CTCAGACGGGGGTCAGAGT | CCACCTCGAAGCTTTCTCAT |
| 2nd PCR | MSTN | 2 | ACACTCTTTCCCTACACGAC GCTCTTCCGATCT TGGAAGGAAAACCCAAATGT | GTGACTGGAGTTCAGACGTGT GCTCTTCCGATCT CCTGGGTTCATGTCAAGTTTC |
| | MSTN Off-target 1 | 2 | ACACTCTTTCCCTACACGAC GCTCTTCCGATCT CTCAGCACTCCAGAGTCCAA | GTGACTGGAGTTCAGACGTGT GCTCTTCCGATCT CCCCATGAGTCTGATAATAGGC |
| | MSTN Off-target 2 | 4 | ACACTCTTTCCCTACACGAC GCTCTTCCGATCT TCCACCCTGGAGATTCTGAC | GTGACTGGAGTTCAGACGTGT GCTCTTCCGATCT GCTAGGAATGTTGAGGCGTTA |
| | MSTN Off-target 3 | 8 | ACACTCTTTCCCTACACGAC GCTCTTCCGATCT TGTGTGTGAGTTCTCAAGTGTAAAA | GTGACTGGAGTTCAGACGTGT GCTCTTCCGATCT GCCAAGAGAAAAAGAGAGAGAAA |
| | MSTN Off-target 4 | 9 | ACACTCTTTCCCTACACGAC GCTCTTCCGATCT GGGCTCCTATCCCTTTACCA | GTGACTGGAGTTCAGACGTGT GCTCTTCCGATCT ACGGGAAGAGGTCTCCGTAA |
| | MSTN Off-target 5 | 20 | ACACTCTTTCCCTACACGAC GCTCTTCCGATCT CACTTCCAATTTGCCCTGAT | GTGACTGGAGTTCAGACGTGT GCTCTTCCGATCT TGAAAGAGGAACTCCCAAGG |
| | PRNP | 13 | ACACTCTTTCCCTACACGAC GCTCTTCCGATCT GCAACCGTTATCCACCTCAG | GTGACTGGAGTTCAGACGTGT GCTCTTCCGATCT CCAGCATGTAGCCACCAAG |
| | PRNP Off-target 1 | 3 | ACACTCTTTCCCTACACGAC GCTCTTCCGATCT CATGTTTAAGCTCAGAAATATGGA | GTGACTGGAGTTCAGACGTGT GCTCTTCCGATCT TTTCATTAATTTGGAGTGATTCTCTT |
| | PRNP Off-target 2 | 5 | ACACTCTTTCCCTACACGAC GCTCTTCCGATCT CAGCCCCCTCATAAGTGTGT | GTGACTGGAGTTCAGACGTGT GCTCTTCCGATCT CCCTCCAAAGCCTTGTTCTT |
| | PRNP Off-target 3 | 7 | ACACTCTTTCCCTACACGAC GCTCTTCCGATCT GGAGATGGTGAAGGACAGGA | GTGACTGGAGTTCAGACGTGT GCTCTTCCGATCT GGGGTGTTGCTCCCTAGAAT |
| | PRNP Off-target 4 | 19 | ACACTCTTTCCCTACACGAC GCTCTTCCGATCT CCAGAGAAAAGCCATGCAAT | GTGACTGGAGTTCAGACGTGT GCTCTTCCGATCT AGTCAATGGCAACTGGAAGG |
| | PRNP Off-target 5 | 24 | ACACTCTTTCCCTACACGAC GCTCTTCCGATCT GGCTGTTTTGATATGGGTCA | GTGACTGGAGTTCAGACGTGT GCTCTTCCGATCT GGACAGCTAAAGGCTGTGGA |
| | BLG | 11 | ACACTCTTTCCCTACACGAC GCTCTTCCGATCT GCTGTCTTTCAGGGAGAACG | GTGACTGGAGTTCAGACGTGT GCTCTTCCGATCT CACGGCAGTGTCTTCATCAC |
