# Supplementary material for: Generation of double knockout cattle via CRISPR-Cas9 ribonucleoprotein (RNP) electroporation
Source: J Anim Sci Biotechnol. 2023 Aug 6;14:103. doi: 10.1186/s40104-023-00902-8 (PMC10404370; doi:10.1186/s40104-023-00902-8)
Supplement: Supplementary file 3 — Additional file 3. Off-target effect detection for gene edited calves. [file 40104_2023_902_MOESM3_ESM.pptx]

## Slide 1
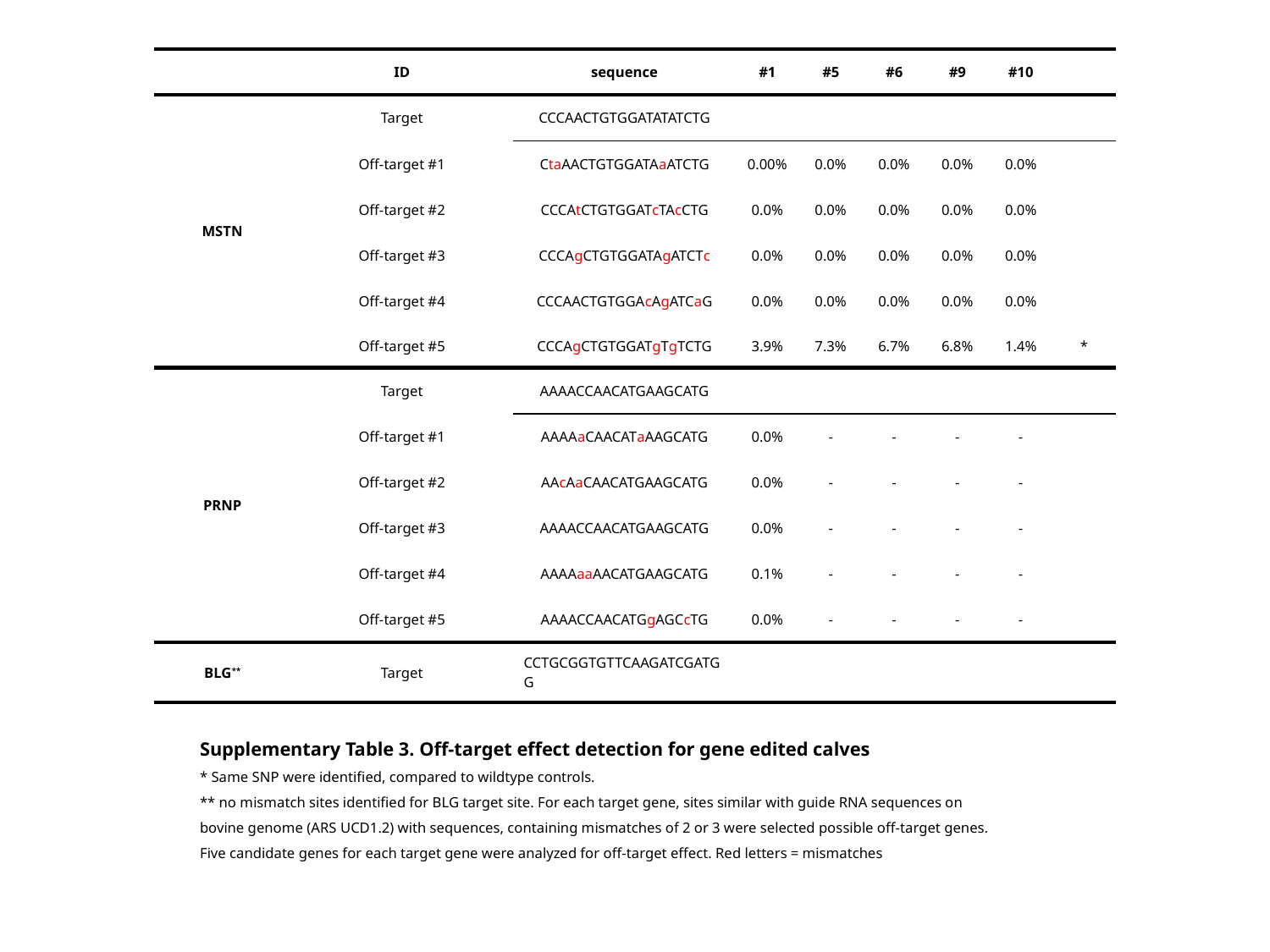

| | ID | sequence | #1 | #5 | #6 | #9 | #10 | |
| --- | --- | --- | --- | --- | --- | --- | --- | --- |
| MSTN | Target | CCCAACTGTGGATATATCTG | | | | | | |
| | Off-target #1 | CtaAACTGTGGATAaATCTG | 0.00% | 0.0% | 0.0% | 0.0% | 0.0% | |
| | Off-target #2 | CCCAtCTGTGGATcTAcCTG | 0.0% | 0.0% | 0.0% | 0.0% | 0.0% | |
| | Off-target #3 | CCCAgCTGTGGATAgATCTc | 0.0% | 0.0% | 0.0% | 0.0% | 0.0% | |
| | Off-target #4 | CCCAACTGTGGAcAgATCaG | 0.0% | 0.0% | 0.0% | 0.0% | 0.0% | |
| | Off-target #5 | CCCAgCTGTGGATgTgTCTG | 3.9% | 7.3% | 6.7% | 6.8% | 1.4% | \* |
| PRNP | Target | AAAACCAACATGAAGCATG | | | | | | |
| | Off-target #1 | AAAAaCAACATaAAGCATG | 0.0% | - | - | - | - | |
| | Off-target #2 | AAcAaCAACATGAAGCATG | 0.0% | - | - | - | - | |
| | Off-target #3 | AAAACCAACATGAAGCATG | 0.0% | - | - | - | - | |
| | Off-target #4 | AAAAaaAACATGAAGCATG | 0.1% | - | - | - | - | |
| | Off-target #5 | AAAACCAACATGgAGCcTG | 0.0% | - | - | - | - | |
| BLG\*\* | Target | CCTGCGGTGTTCAAGATCGATGG | | | | | | |
Supplementary Table 3. Off-target effect detection for gene edited calves
* Same SNP were identified, compared to wildtype controls.
** no mismatch sites identified for BLG target site. For each target gene, sites similar with guide RNA sequences on bovine genome (ARS UCD1.2) with sequences, containing mismatches of 2 or 3 were selected possible off-target genes. Five candidate genes for each target gene were analyzed for off-target effect. Red letters = mismatches
