# Supplementary material for: Generation of double knockout cattle via CRISPR-Cas9 ribonucleoprotein (RNP) electroporation
Source: J Anim Sci Biotechnol. 2023 Aug 6;14:103. doi: 10.1186/s40104-023-00902-8 (PMC10404370; doi:10.1186/s40104-023-00902-8)
Supplement: Supplementary file 4 — Additional file 4. Blood analysis of calves with gene mutation. [file 40104_2023_902_MOESM4_ESM.pptx]

## Slide 1
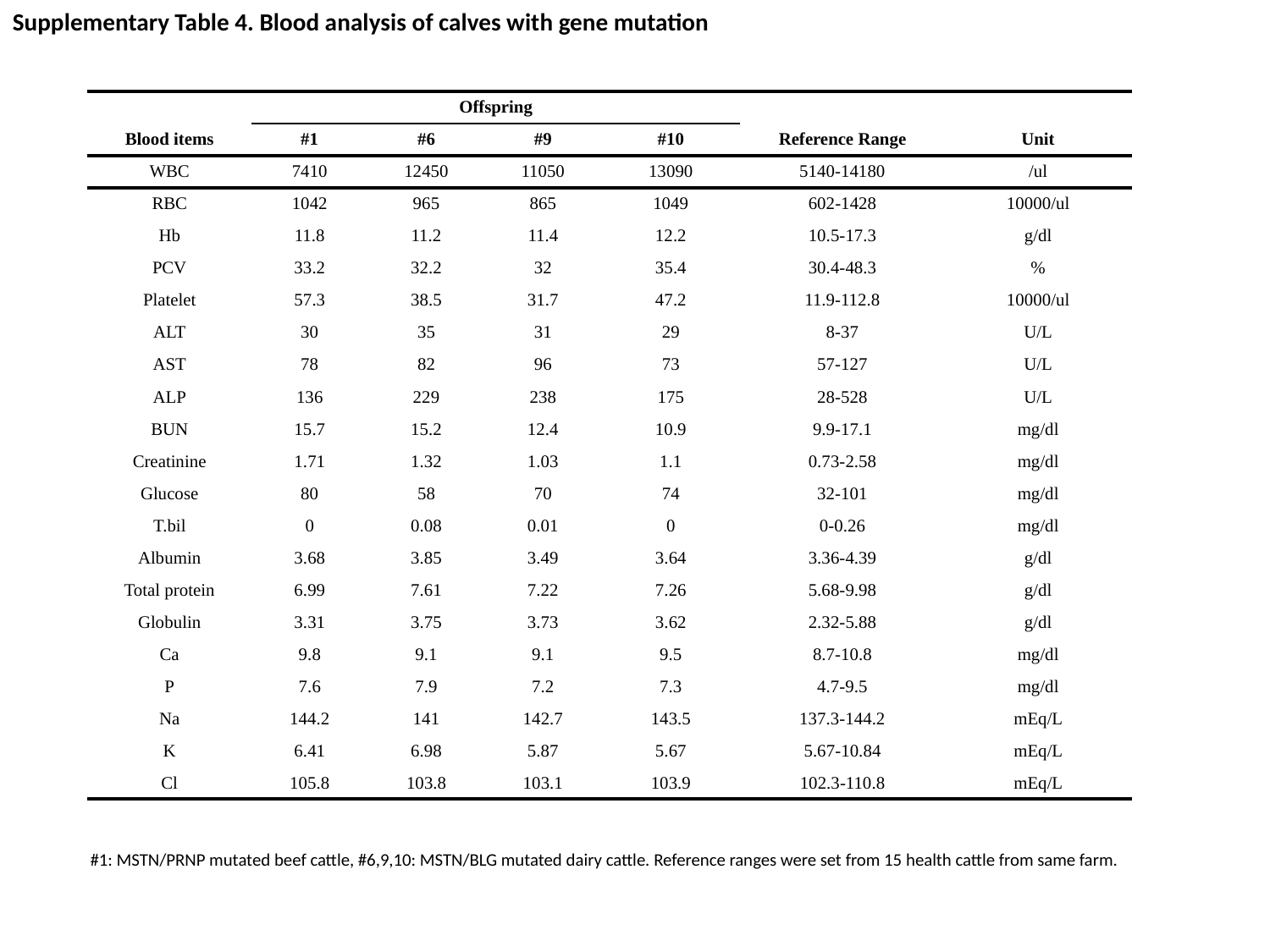

Supplementary Table 4. Blood analysis of calves with gene mutation
| | Offspring | | | | | |
| --- | --- | --- | --- | --- | --- | --- |
| Blood items | #1 | #6 | #9 | #10 | Reference Range | Unit |
| WBC | 7410 | 12450 | 11050 | 13090 | 5140-14180 | /ul |
| RBC | 1042 | 965 | 865 | 1049 | 602-1428 | 10000/ul |
| Hb | 11.8 | 11.2 | 11.4 | 12.2 | 10.5-17.3 | g/dl |
| PCV | 33.2 | 32.2 | 32 | 35.4 | 30.4-48.3 | % |
| Platelet | 57.3 | 38.5 | 31.7 | 47.2 | 11.9-112.8 | 10000/ul |
| ALT | 30 | 35 | 31 | 29 | 8-37 | U/L |
| AST | 78 | 82 | 96 | 73 | 57-127 | U/L |
| ALP | 136 | 229 | 238 | 175 | 28-528 | U/L |
| BUN | 15.7 | 15.2 | 12.4 | 10.9 | 9.9-17.1 | mg/dl |
| Creatinine | 1.71 | 1.32 | 1.03 | 1.1 | 0.73-2.58 | mg/dl |
| Glucose | 80 | 58 | 70 | 74 | 32-101 | mg/dl |
| T.bil | 0 | 0.08 | 0.01 | 0 | 0-0.26 | mg/dl |
| Albumin | 3.68 | 3.85 | 3.49 | 3.64 | 3.36-4.39 | g/dl |
| Total protein | 6.99 | 7.61 | 7.22 | 7.26 | 5.68-9.98 | g/dl |
| Globulin | 3.31 | 3.75 | 3.73 | 3.62 | 2.32-5.88 | g/dl |
| Ca | 9.8 | 9.1 | 9.1 | 9.5 | 8.7-10.8 | mg/dl |
| P | 7.6 | 7.9 | 7.2 | 7.3 | 4.7-9.5 | mg/dl |
| Na | 144.2 | 141 | 142.7 | 143.5 | 137.3-144.2 | mEq/L |
| K | 6.41 | 6.98 | 5.87 | 5.67 | 5.67-10.84 | mEq/L |
| Cl | 105.8 | 103.8 | 103.1 | 103.9 | 102.3-110.8 | mEq/L |
#1: MSTN/PRNP mutated beef cattle, #6,9,10: MSTN/BLG mutated dairy cattle. Reference ranges were set from 15 health cattle from same farm.
